# MRI Evaluation of Rectal Cancer Lymph Node Staging Using Apparent Diffusion Coefficient

**DOI:** 10.7759/cureus.45002

**Published:** 2023-09-10

**Authors:** Ingrida Pikūnienė, Žilvinas Saladžinskas, Algidas Basevičius, Vestina Strakšytė, Justas Žilinskas, Rita Ambrazienė

**Affiliations:** 1 Department of Radiology, Hospital of Lithuanian University of Health Sciences Kauno Klinikos, Kaunas, LTU; 2 Department of Surgery, Hospital of Lithuanian University of Health Sciences Kauno Klinikos, Kaunas, LTU; 3 Department of Oncology, Hospital of Lithuanian University of Health Sciences Kauno Klinikos, Kaunas, LTU

**Keywords:** apparent diffusion coefficient, diffusion-weighted imaging, magnetic resonance imaging, lymph nodes, rectal cancer

## Abstract

Introduction

Colorectal cancer is the third most diagnosed cancer globally. Lymph node metastases significantly affect prognosis, emphasizing the importance of early detection and management. Despite significant advances in conventional MRI's role in staging, improvements in advanced functional imaging such as diffusion-weighted imaging (DWI) in identifying lymph node metastases persist.

Objectives

The aim is to evaluate the effectiveness of apparent diffusion coefficient (ADC) MRI in evaluating lymph node staging in rectal cancer.

Patients and methods

In a prospective study, 89 patients with stage II-III rectal cancer were grouped into two treatments: pre-operative FOLFOX4 chemotherapy and standard pre-operative chemoradiotherapy. All underwent 1.5T MRI, with T2-weighted and DWI sequences. A radiologist defined regions of interest on the tumor, lymph nodes, and intact rectal wall to calculate ADC values.

Results

Rectal cancer ADC's receiver operating characteristic curve had an area under the curve (AUC) of 0.688 (P < 0.001), with optimal ADC cutoff at 0.99 x 10-3 mm2/s (sensitivity: 75%, specificity: 83%). For lymph nodes, AUC was 0.508 (P < 0.001), with a cutoff of 0.9 x 10-3 mm2/s (sensitivity: 78%, specificity: 67%). No correlation between tumor and lymph node ADC values was observed. In chemotherapy patients, "healthy" inguinal lymph nodes had higher ADC values than affected ones pre-treatment (P = 0.001), a disparity fading post-treatment (P = 0.313). For chemoradiotherapy patients, the ADC difference persisted pre and post-treatment (P = 0.001).

Conclusion

The study of ADC-MRI showed different ADC values between tumors and lymph nodes and highlighted ADC differences between treatment groups. Notably, no correlation was observed between tumor and lymph node ADC values. However, differences were apparent when comparing "healthy" inguinal nodes with lymph nodes affected by cancer.

## Introduction

Colorectal cancer is a major global health issue with a high incidence and mortality rate. As the third most common cancer globally and the second in terms of cancer-related deaths, it substantially burdens healthcare systems and society as a whole [1]. It is the third most common cancer in men and the second most common cancer in women. There were more than 1.9 million new cases of colorectal cancer in 2020 [2]. In Europe, colorectal cancer ranks among the top cancer types, affecting both men and women, making it a critical health issue for the region.

Locally advanced colorectal cancer (LACRC) constitutes a notable portion of colorectal cancer cases, accounting for approximately 10-15% [3,4]. The prevalence of LACRC tends to be higher in more developed countries, likely because of lifestyle, diet, and aging populations. However, these countries also demonstrate a lower mortality rate, which can be attributed to improved screening practices and advancements in rectal cancer staging and treatment. Early detection and appropriate management have improved patient outcomes [5].

An emerging concern is the rising prevalence of colorectal cancer among younger individuals, particularly those below the age of 50 years. This trend has been observed in recent years, and the death rate among this age group has also increased by 1% per year [6]. Identifying the reasons behind this alarming trend and implementing effective preventive measures are critical to addressing this growing public health issue and improving survival rates in younger colorectal cancer patients.

Lymph node metastases play a pivotal role in forecasting the outcome of rectal cancer. Earlier research has demonstrated that patients with pN2 node involvement are more likely to have a shorter lifespan [6,7]. Therefore, identifying and determining the extent of lymph node metastasis is essential for ensuring accurate diagnosis and deciding the adjuvant chemotherapy (CT) requirement after surgical resection [7]. For rectal cancer, neoadjuvant (i.e., pre-operative) instead of adjuvant (i.e., post-operative) chemoradiotherapy (CRT) has become the standard of care in the United States and Europe after the German rectal cancer trial, which demonstrated better local control and reduced toxicity with neoadjuvant CRT compared with adjuvant CRT [8]. Several smaller studies found similar results in patients with advanced colon cancer [9,10]. The decision of whether a patient with rectal cancer is a candidate for total mesorectal excision (TME) only or neoadjuvant therapy followed by TME is made on the findings of MRI. MRI of rectal cancer patients offers promising results considering T-staging because of the high contrast resolution for the soft tissues, allowing the best depiction of neoplastic lesions and their anatomical relationships [10]. Therefore, MRI examination is considered the gold standard for locoregional staging and restaging in rectal cancer, according to the main international guidelines [11]. However, MRI is less accurate in N staging than in tumor (T) staging, with sensitivity and specificity values ranging between 58-77% and 62-74%, respectively [12]. Unfortunately, accurate N staging (i.e., prediction of lymph node metastasis) is an important prognostic factor and indicator for using neoadjuvant CRT, which remains a problem [13-15].

Unfortunately, previous research has shown that MRI has limited effectiveness in accurately identifying metastatic lymph nodes [16]. Presently, there are diverse diagnostic criteria for metastatic lymph nodes, including considerations of size, shape, and delineation, but a consensus on the precise diagnosis still needs to be achieved [17].

Past studies have suggested that diffusion-weighted imaging (DWI) may be more suitable for tumor detection and identification than conventional MRI. This advantage could be attributed to DWI’s ability to reflect specific histopathological differences between metastatic and non-metastatic lymph nodes, possibly because of its association with cellularity and microvasculature [18-20].

DWI-MRI, a functional MRI component, can be used to investigate meaningful biological properties such as tissue cellularity and water content. The apparent diffusion coefficient (ADC) provides valuable information about tissue properties and image contrast. ADC serves as a measure of diffusion in biological systems and has proven to be a quantitative biomarker with various applications [21,22].

## Materials and methods

Patients

The study included patients who attended Lithuanian Health Science University Kaunas Clinics between 2015 and 2022 to treat rectal tumors diagnosed with stage II-III rectal cancer. This was a prospective single-institution clinical trial (ClinicalTrials.gov Identifier: NCT05378919), and the Regional Biomedical Research Ethics Committee approved the study (Protocol No.: BE-2-32). All patients provided informed consent.

A total of 89 patients, 60 men and 29 women, with an average age of 64 years (range from 37 to 83), were included in the final study.

All enrolled patients met all inclusion criteria and none of the exclusion criteria. Inclusion criteria were age (>18 years), biopsy-proven adenocarcinoma of the rectum (0-15 cm till above anal verge), radiologically measurable tumor size, II-III rectal cancer stage, Eastern Cooperative Oncology Group (ECOG) 0-2, and no distant metastasis. Exclusion criteria were patients with signs of intestinal obstruction at the beginning of treatment, previous radiation therapy in the lower abdomen, another tumor within five years, pregnant or nursing women, and patient’s comorbidities that would make the patient ineligible for this study or significantly interfere with safety and toxicity evaluation.

The study participants were split into two treatment cohorts at a 1:1 ratio, with 40 patients in the CT group and 49 patients in the CRT group. The initial cohort was designated to undergo the new standard pre-operative FOLFOX4 CT regimen, comprising eight cycles. The efficacy of the treatment was evaluated using radiological techniques (pelvis MRI) after the evaluation of cancer marker dynamics and the feasibility of conducting radical surgery (geared toward achieving an R0 procedure). Supplementary fractional radiation therapy was introduced if the tumor sustained its T4 dimensions or exhibited N (+) status based on the TNM (tumor, node, and metastasis) classification during the pre-operative phase. Total dose (TD) of 50 Gy at five radiations per week of 2 Gy for five weeks, along with an additional two courses of 5-Fu/folic acid chemotherapy (fluorouracil/leucovorin 400 mg/m² for one to four days at the first and fifth weeks of radiotherapy) were given. In the event of tumor size reduction (T1-3, N0), patients proceeded with surgical intervention. Post-surgery, CT was scheduled to persist with an additional four cycles (completing six months of systemic therapy), following the FOLFOX4 protocol, contingent on achieving R0 surgery. The second group, the control group, underwent standard CRT during the pre-operative phase and postoperatively (four cycles of chemotherapy in the Mayo regimen).

MRI protocol

MRI was performed using a 1.5-T scanner (Magnetom Avanto, Siemens Healthcare, Erlangen, Germany) with phased-array surface coils. The patient was in the supine position, and the surface coil was placed on the pelvis. Patients underwent pre-treatment pelvic MRI for tumor staging and restaging MRI for assessment of response six to eight weeks after treatment (CT alone or standard neoadjuvant CRT).

Before conducting the scans, all patients underwent a fasting period of four to six hours and underwent bowel preparation involving micro enema administration within 0.5-2 hours preceding the MRI scan. An intravenous bolus injection of 20 mg of butylscopolamine (Buscopan®, Boehringer Ingelheim B.V., Ingelheim, Germany) was administered to diminish peristaltic movement. For all subsequent restaging MRI examinations, a contrast-enhanced approach was adopted, entailing the injection of 0.2 mL/kg of Gd-based contrast media at a rate of 3.0 mL/s, followed by a saline infusion of 20 mL at the same rate of administration.

The standard imaging protocol included standard two-dimensional T2-weighted (T2W) fast spin-echo sequences in three orthogonal directions (with the transverse images angled perpendicular and the coronal images angled parallel to the tumor axis as identified on the sagittal scan) and an axial echo planar imaging (EPI). The DWI sequence was angled in the same plane as the T2W transverse images. The DWI sequence was performed with spectral attenuated inversion recovery (SPAIR) fat suppression (b values 0, 500, 1000 s/mm2; TR/TE 4147/66 ms; EPI factor 77; five number of signals acquired; 1.82 × 2.26 × 5.00 mm acquisition voxel size, 20 slices, slice gap of 0.5 mm; acquisition time of 6:44 min). ADC maps of isotropic images were created automatically by the device.

MRI ADC evaluation and measurement

**Figure 1:**
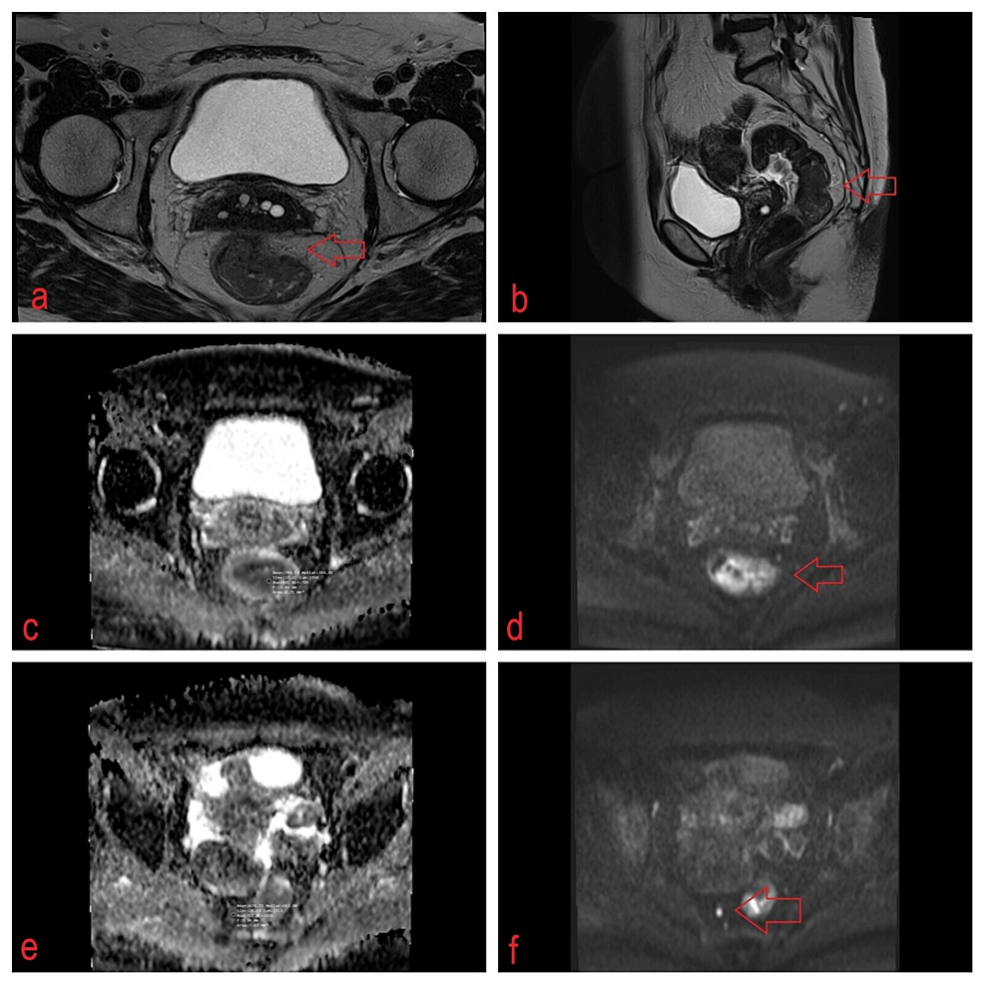
Rectal cancer staged as T3bN2 involves mesorectal fascia and is associated with multiple enlarged mesorectal lymph nodes. Rectal cancer ADC value was 0.748 * 10^−3^ mm^2^/s; suspicious lymph node ADC value was 0.678 * 10^−3 ^mm^2^/s. (a) Axial T2; (b) Sag T2; (c) rectal cancer ADC map and (e) lymph node ADC map; (d) rectal cancer high-b value (b = 1000 s/mm^2^) DWI image and (f) lymph node high-b value (b=1000 s/mm^2^) DWI image. ADC: apparent diffusion coefficient; DWI: diffusion-weighted imaging.

A radiologist with seven years of experience interpreting rectal MRI scans conducted the assessment without knowing the pathological results. He manually outlined the regions of interest (ROIs) on successive ADC map tumor slices (b = 1000 s/mm2). The mean ADC of the rectal mass was computed by selecting three different areas of interest (ROIs) within the tumor across the images. These areas were identified as restricted regions on the ADC map corresponding to their isotropic DWI, and the average ADC value was then calculated. T2W sequences were used to locate the primary tumor and lymph nodes. Each ROI corresponding to the maximum cross-sectional tumor size on the ADC map was delineated, effectively avoiding the T2W shine-through effect. This approach helped achieve clear demarcation between the tumor and normal tissue (Figure 1).

Statistical analysis

Clinical, laboratory, and outcome measurement data were coded, recorded, and evaluated using Microsoft Excel (Microsoft Corporation, Redmond, WA). Statistical analysis was performed using SPSS for Windows version 23.0 software (IBM Corp., Armonk, NY) and Microsoft Excel 16™. Several tests were used: chi-square test (X2), kappa test, t-test, ANOVA, and Pearson’s correlation coefficient. P-value was established at <0.05 for significant results.

## Results

Our study included 89 patients; their ages ranged from 37 to 83 years, with a mean age of 64.26 (SD 9.165) years. Most patients were males (60, 67.4%), while females were 29 (32.6%). Tumor staging was assessed via imaging scans (MRI T2W and DWI) and pathological examinations (Table 1). Within the CT group, there were 67.5% (27) males and 32.5% (13) females, whereas there were 67.3% (33) males and 32.6% (16) females in the control CRT group.

**Table 1:** Tumor stage, clinical, radiological, and pathological distribution in the chemotherapy and chemoradiotherapy groups. c = clinical; y = after treatment; cT = clinical stage before treatment; yT = stage after treatment; pT = pathological stage; N = nodal staging; EMVI = extramural venous invasion; LVI = lymphovascular invasion (pathological); MRF = mesorectal fascia; CRM = circumferential resection margin; CEA = carcinoembryonic antigen; CA19-9 = carbohydrate antigen 19-9; SD = standard deviation.

	Chemotherapy (CT) group (n = 40; % in group)			Control chemoradiotherapy (CRT) group (n = 49; % in group)				
MRI T stage		cT	yT	cT	yT			
	T0	-	2 (5)	-	4 (8.2)			
	T2	-	6 (15)	3 (6.1)	-			
	T3a	-	10 (25)	19 (38.8)	8 (16.3)			
	T3b	16 (40)	19 (47.5)	14 (28.5)	37 (75.5)			
	T3c	13 (32.5)	2 (5)	7 (14.3)	-			
	T3d	5 (12.5)	1 (2.5)	6 (12.2)	-			
	T4a	6 (15.0)	-	-	-			
pT stage	T0	4 (10)		9 (18.4)				
	T1	1 (2.5)		2 (4.1)				
	T2	8 (20)		9 (18.4)				
	T3	27 (67.5)		29 (59.2)				
N stage		cN	yN	cN		yN		
	N0	-	15 (37.5)	1 (2)		19 (38.8)		
	N1	13 (32.5)	11 (27.5)	17 (34.7)		27 (55.1)		
	N2	27 (67.5)	14 (35.0)	31 (63)		3 (6.1)		
pN stage	N0	21 (52.5)		33 (67.3)				
	N1	15 (37.5)		12 (24.5)				
	N2	4 (10)		4 (8.2)				
EMVI (radiological)		cEMVI	yEMVI	cEMVI			yEMVI	
	EMVI-	17 (42.5)	28 (70)	27 (55.1)			34 (69.4)	
	EMVI+	23 (57.5)	12 (30)	22 (44.9)			15 (30.6)	
LVI (pathological)	LVI-	24 (60)		34 (69.4)				
	LVI+	16 (40)		15 (30.6)				
MRF		cMRF	yMRF	cMRF			yMRF	
	MRF-	20 (50)	28 (70)	20 (40.8)			38 (77.6)	
	MRF+	20 (50)	12 (30)	29 (59.2)			11 (22.4)	
CRM (after resection)	CRM-	35 (87.5)		46 (93.9)				
	CRM+	5 (12.5)		3 (6.1)				
	Before treatment		After treatment	Before treatment				After treatment
CEA	10.19 (SD 15.54)		3.34 (SD 2.49)	8.48 (SD 10.37)				4.1 (SD 6.11)
CA-19.9	20.46 (SD 23.53)		14.79 (SD 11.58)	18.46 (SD 16.53)				22.98 (SD 73.69)

At the time of initial diagnosis, MRI scans of patients within the CT group revealed that 16 patients (40.0%) were classified as stage T3b, whereas five patients (12.5%) were identified as stage T3d.

After undergoing FOLFOX treatment, significant changes in disease staging were observed. Specifically, two patients (5.0%) showed no evidence of the disease and were classified as T0. In addition, 19 patients (47.5%) were categorized as stage T3b. This change was statistically significant, with a P-value of 0.001. Alternatively, when broadly categorizing the results, two patients (5.0%) were at T0, and a considerable majority (32 patients, 80.0%) were at the T3 stage.

Within the control CRT group, initial MRI scans before treatment indicated that three patients (6.1%) were classified as T2, whereas 19 patients (38.8%) fell into the T3a category. Post-treatment results displayed significant changes: four patients (8.2%) showed no trace of the disease and were classified as T0, and 37 patients (75.5%) were identified as T3b. When evaluated statistically, this transition had a P-value of 0.060, indicating borderline significance. When summarizing the post-treatment stages more broadly, four patients (8.2%) were at T0, whereas a substantial 45 patients (91.8%) were categorized as T3.

The pathological analysis results varied between the CT and control CRT groups. Specifically, within the CT group, the confirmed pathological stages were as follows: four patients (10.0%) were classified as T0, one patient (2.5%) as T1, eight patients (20.0%) as T2, and most (27, 67.5%) patients as T3. This distribution was found to be statistically significant with a P-value of 0.008. On the other hand, in the control CRT group, nine (18.4%) patients were found to be T0, two (4.1%) patients were T1, nine patients (18.4%) were T2, and 29 patients (59.2%) were T3, showing a P-value of 0.001, which emphasizes a highly significant variation in their distribution.

In the CT group, the staging was as follows: initial: 32.5% as N1 and 67.5% as N2; after treatment: 37.5% N0, 27.5% N1, and 35% N2; pathologically confirmed: 52.5% N0, 37.5% N1, and 10% N0. In the CRT group, N staging was initially referred to as 2% as N0, 34.7% as N1, and 63% as N2; after treatment: 38.8% N0, 55.1% N1, and 6.1% N2; pathologically confirmed: 67.3% N0, 24.5% N1, and 8.2% N0. In both groups, treatment significantly reduced N2 staging, as confirmed pathologically (P = 0.001).

**Figure 2:**
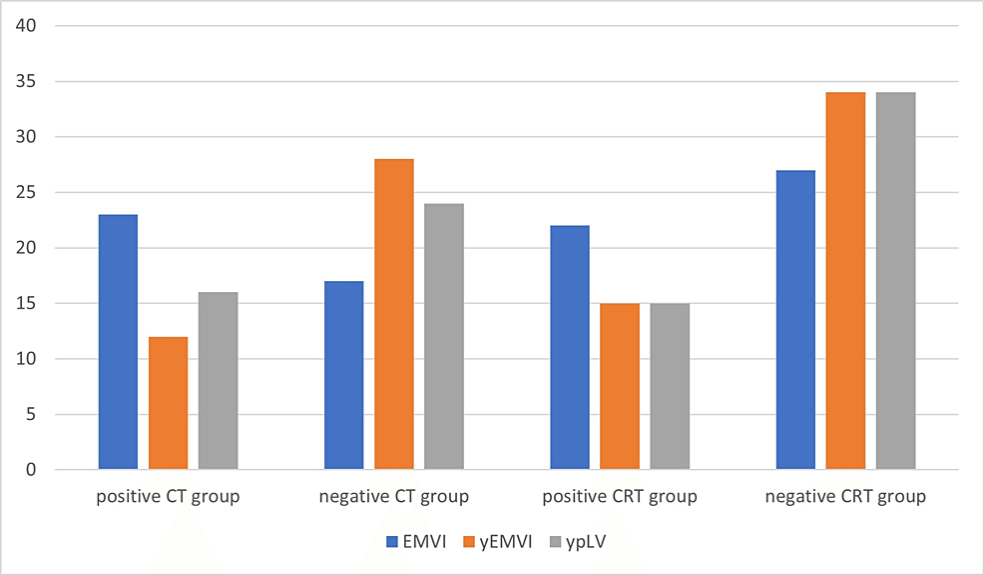
Diagram of both treatment groups according to EMVI and lymphovascular invasion changes. Positive CT/CRT group = extramural venous invasion was found. Negative CT/CRT group = no extramural venous invasion was found. ypLV = pathology proven lymphovascular invasion. CT = chemotherapy; CRT = chemoradiotherapy; EMVI = extramural venous invasion before treatment; yEMVI = extramural venous invasion after treatment; ypLV = lymphovascular invasion proven by pathology.

Both the CT and CRT treatment groups showed an increase in the percentage of patients with negative lymphovascular/extramural venous invasion after treatment. The change was statistically significant (P = 0.001) for the CT group but not for the CRT group (P = 0.065). However, when comparing the post-treatment pathological analysis of both groups, there was no significant difference in EMVI status (P = 0.219 in CT and P = 1.00 in CRT groups) (Figure 2).

**Figure 3:**
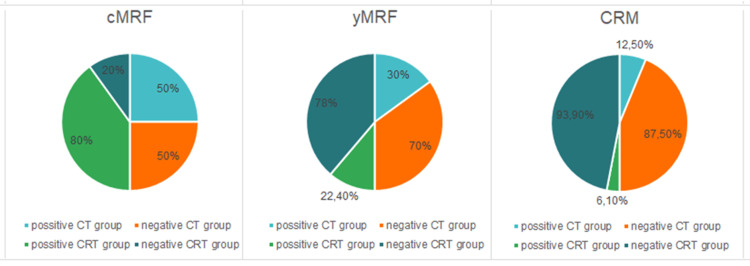
Diagram of both treatment groups according to the mesorectal fascia changes. Positive* CT/CRT group = involved mesorectal fascia or circumferential resection margin. Negative* CT/CRT group = no mesorectal fascia or circumferential resection margin involvement by the tumor. CT = chemotherapy; CRT = chemoradiotherapy; cMRF = mesorectal fascia before treatment; yMRF = mesorectal fascia after treatment; CRM = circumferential resection margin proven by pathology.

Both treatment groups demonstrated an increase in the percentage of patients with mesorectal fascia (MRF) negative (without MRF involvement) after treatment, with the change being statistically significant for both groups (P = 0.001). Furthermore, the pathological analysis showed a higher proportion of circumferential resection margin (CRM)-negative patients in both groups post-treatment, with the differences being statistically significant (P = 0.016 and P = 0.021, respectively, to CT and CRT groups) (Figure 3).

**Figure 4:**
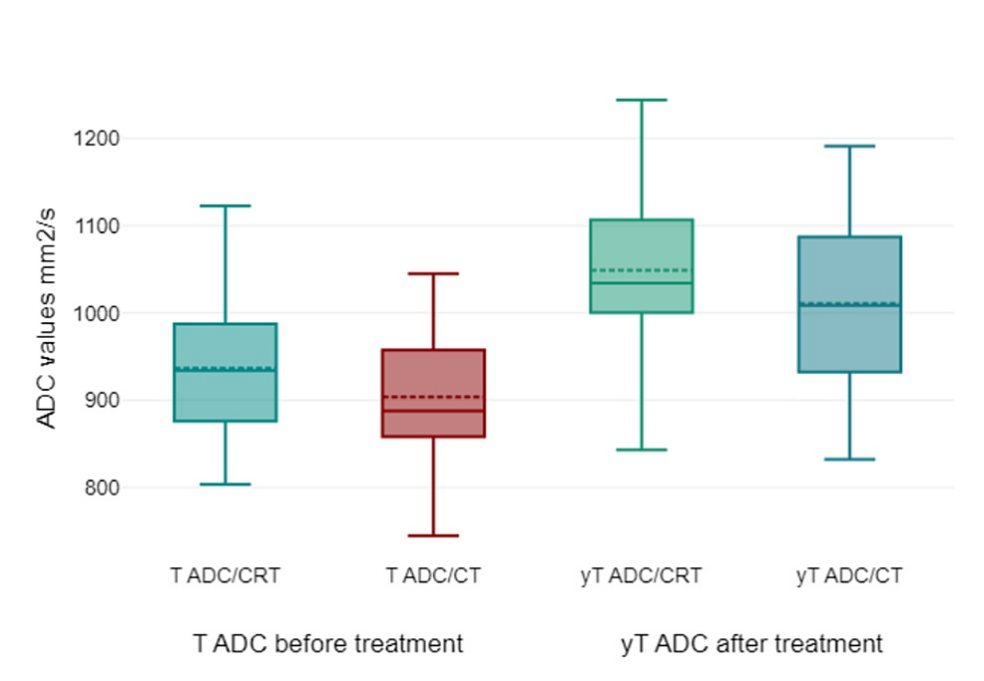
Boxplots for the mean ADC for tumors pre- and post-treatment within both treatment groups. ADC values are given x 10-2 mm2/s. ADC = apparent diffusion coefficient; T ADC/CRT = tumor ADC chemoradiotherapy group/pre-treatment; T ADC/CT = tumor ADC chemotherapy group/pre-treatment; yT ADC/CRT = tumor ADC chemoradiotherapy group/after treatment; yT ADC/CT = tumor ADC chemotherapy group/after treatment. ADC values are given x 10^-2^ mm^2^/s.

**Figure 5:**
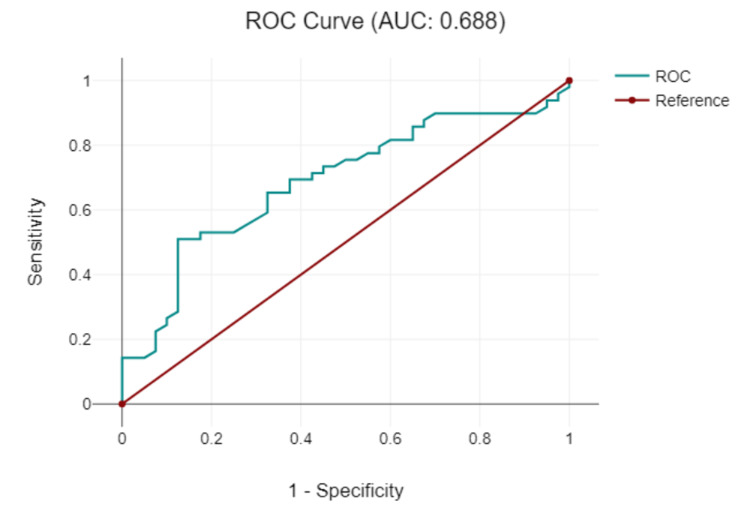
The receiver operating characteristics (ROC) curve of the apparent diffusion coefficient for rectal cancer. AUC: area under the curve.

According to our study, the mean tumor ADC before treatment for all patients was 699.75 (SD 95.35) x 10^-2^ mm^2^/s in the CT group and 768.88 (SD 114.57) x 10^-2^ mm^2^/s in the CRT group. Before any treatment, the CRT group presented with a slightly higher mean ADC value than the CT group, indicating potential differences in tumor microenvironments or inherent tumor characteristics between the two groups. After treatment, ADC values were 830.35 (SD 105.81) x 10^-2^ mm^2^/s in the CT group and 899.41 (115.97) x 10^-2^ mm^2^/s in the CRT group. Post-treatment ADC values increased for both groups. This suggests enhanced water diffusivity, possibly indicative of tumor cell death or a reduction in cell density, making the tumor environment more permeable. The CRT group maintained a higher ADC, which might suggest a more significant response or a different mechanism of action regarding how the combined therapy affects the tumor (Table 2 and Figure 4). The ROC curve of ADC detection (Figure 5) for rectal cancer detection had an area under the curve (AUC) of 0.688 (P < 0.001) and a cutoff value of 0.885 x 10^-3^ mm^2^/s with a sensitivity of 81.8% and a specificity of 71.4%.

**Table 2:** ADC values in rectal cancer T and N stages. ADC = apparent diffusion coefficient; T ADC = tumor ADC; N ADC = lymph node ADC; RV ADC = "healthy" rectal wall ADC; S LM/IN ADC = "healthy" inguinal lymph node ADC.

Count		Chemotherapy (CT) group (n = 40 patients)		Control chemoradiotherapy (CRT) group (n = 49 patients)	
		Before treatment	After treatment	Before treatment	After treatment
T ADC (x10^-2 ^mm^2^/s)	T0		922.00 (SD 32.52)		974.25 (SD 85.63)
	T2		861.33 (SD 137.00)	709.64 (SD 69.92)	
	T3a		837.10 (SD 75.44)	791.21 (SD 118.03)	840.38 (SD 138.46)
	T3b	705.38 (SD 29.44)	825.95 (SD 108.41)	765.14 (SD 114.89)	904.08 (SD 110.32)
	T3c	663.39 (SD 17.95)	751.00 (SD 72.12)	756.43 (SD 148.31)	
	T3d	723.00 (SD 35.38)	636.00 (SD 00.00)	751.00 (SD 92.45)	
	T4a	744.17 (SD 36.01)			
T ADC total (x10^-2 ^mm^2^/s)		699.75 (SD 95.35)	830.35 (SD 105.81)	768.88 (SD 114.57)	899.41 (SD 115.97)
N ADC (x10^-2 ^mm^2^/s)	N0		849.80 (SD 112.71)	879.00 (SD 00.00)	911.21 (SD 125.37)
	N1	688.00 (SD 106.51)	828.36 (SD117.74)	738.59 (SD 132.30)	888.96 (SD 108.88)
	N2	705.41 (SD 91.09)	811.07 (SD 91.66)	781.94 (SD 102.80)	918.67 (SD 154.37)
N ADC total (x10^-2 ^mm^2^/s)		739.33 (SD 71.83)	955.38 (SD 128.74)	744.39 (SD 71.83)	984.78 (SD 73.22)
RV ADC (x10^-2 ^mm^2^/s)		1228.27 (SD 124.82)	1227.67 (SD 169.29)	1219.80 (SD 128.44)	1219.80 (SD 148.13)
S LM/IN ADC (x10^-2 ^mm^2^/s)		844.05 (SD 102.02)	976.20 (SD 71.92)	858.94 (SD 104.01)	1140.80 (SD 1263.93)

**Figure 6:**
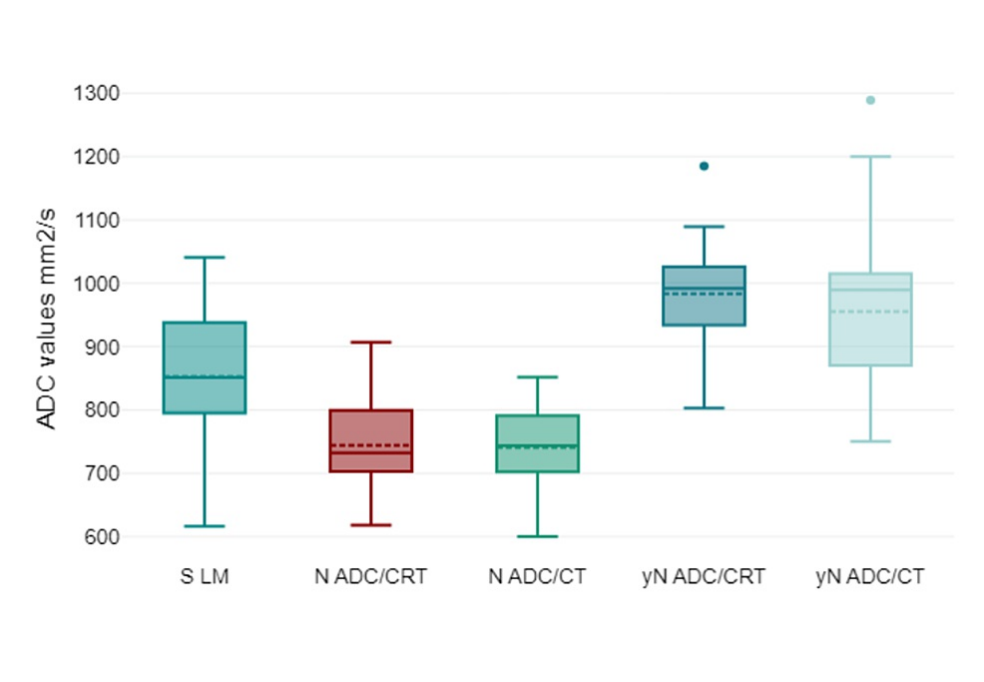
Boxplots for the mean ADC for "healthy" inguinal lymph node "S LM" pre- and after treatment within both treatment groups. ADC values are given in mm2/s x 10-3. ADC = apparent diffusion coefficient; S LM = mean ADC for "healthy" inguinal lymph node; N ADC/CT = lymph node ADC chemotherapy group/pre-treatment; N ADC/CRT = lymph node ADC chemoradiotherapy group/pre-treatment; yN ADC/CT = lymph node ADC chemotherapy group/after treatment; yN ADC/CRT = lymph node ADC chemoradiotherapy group/after treatment. ADC values are given x 10^-2 ^mm^2^/s.

**Figure 7:**
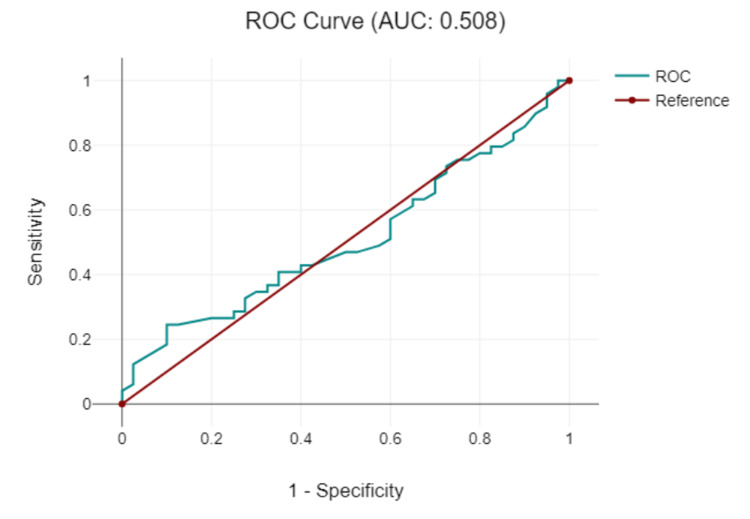
The receiver operating characteristic (ROC) curve of the apparent diffusion coefficient for detecting rectal cancer lymph nodes. AUC: area under the curve.

This study presents the lymph node ADC values, a parameter that provides insights into lymph node cellularity and potential treatment efficacy. Before treatment, both the CT and CRT groups had comparable ADC values, with 739.33 (SD 71.83) x 10^−2^ mm^2^/s in the CT group and a slightly higher 744.39 (SD 71.83) x 10^−2^ mm^2^/s in the CRT group. This close resemblance suggests initial similarities in the cellular environment of the lymph nodes in both groups. However, after treatment, there was a notable increase in the ADC values for both groups, implying changes in the lymph nodes, potentially indicative of a positive therapeutic response. Specifically, the CT group recorded an ADC value of 955.38 (SD 105.81) x 10^−2^ mm^2^/s, whereas the CRT group showed a slightly more elevated ADC value of 984.78 (SD 73.22) x 10^−2^ mm^2^/s. This increase in ADC post-treatment suggests that both treatments might effectively alter the lymph node environment, possibly indicating decreased cellularity, which can predict treatment efficacy. However, the slightly higher ADC value in the CRT group post-treatment could hint at a marginally more pronounced effect or different treatment dynamics in the combined CRT approach compared with CT alone (Table 2 and Figure 6). The ROC curve of lymph node ADC detection (Figure 7) for rectal cancer had an AUC of 0.508 (P < 0.001) and a cutoff value of 0.9 x 10^-3^ mm^2^/s with sensitivity of 78% and specificity of 57%.

**Figure 8:**
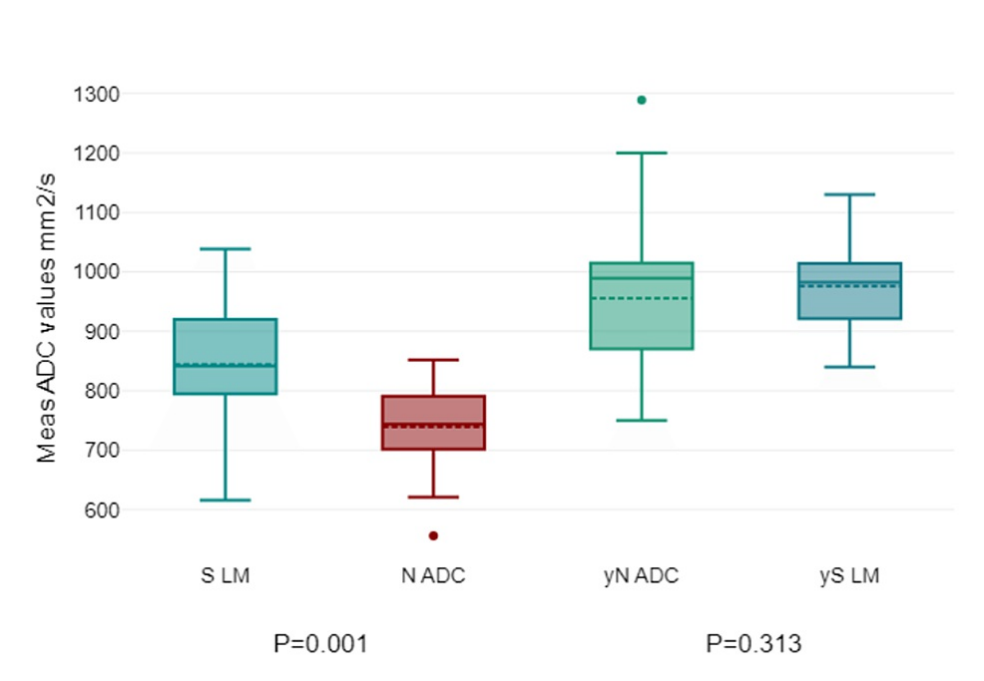
Chemotherapy group's lymph node's ADC. Chemotherapy group: ADC = apparent diffusion coefficient; N ADC = lymph node ADC; S LM = "healthy" inguinal lymph node; yN ADC = lymph node ADC after treatment; yS LM = "healthy" inguinal lymph node after treatment/IN ADC average.

**Figure 9:**
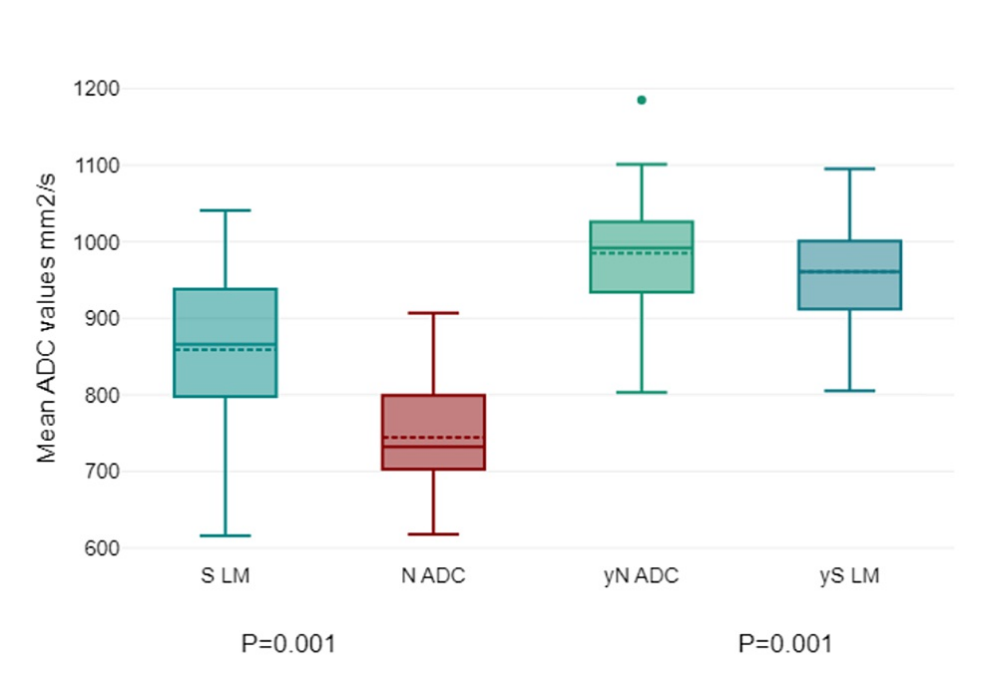
Chemoradiotherapy group's lymph node's ADC. Chemoradiotherapy group: ADC = apparent diffusion coefficient; N ADC = lymph node ADC; S LM = "healthy" inguinal lymph node; yN ADC = lymph node ADC after treatment; yS LM= "healthy" inguinal lymph node after treatment/IN ADC average.

No significant correlation was observed between the minimum, average, or maximum ADC values of the tumor and ADC values of lymph nodes (P > 0.376). However, the pre-treatment "healthy" inguinal lymph node/IN ADC average in the CT group was significantly higher than that in the N ADC (P = 0.001). After treatment, no significant difference between the averages was found (P = 0.313) (Figure 8). Meanwhile, in the CRT group, the "healthy" inguinal lymph node/IN ADC average was significantly higher in both pre-treatment and post-treatment (P = 0.001) groups (Figure 9).

## Discussion

In this study involving 89 patients diagnosed with rectal cancer, there were several key points of interest: the patient cohort was predominantly male, representing approximately 67% of the total. The mean age was 64.26 (SD 9.165) years, ranging from 37 to 83 years. This age distribution and male predominance are consistent with global statistics, which show that rectal cancer incidence increases with age and has a slight male predominance [23].

According to MRI analysis (pre-treatment), most tumor staging and treatment effects in the CT group (FOLFOX treatment) were at stages T3b and T3c, accounting for 40% and 32.5%, respectively. Notably, post-treatment MRI staging showed a marked decrease in the percentage of T3c and T3d stages (from 45% combined to 7.5%). The largest proportion of patients post-treatment was in the T3b stage (47.5%). This suggests that the FOLFOX treatment significantly downstages the tumor in many patients, given the statistical significance (P = 0.001). In MRI analysis (pre-treatment), in the control CRT group, a more diverse distribution was observed, with the majority in T3a (38.8%) and T3b (28.5%) stages. The MRI results show a post-treatment shift majorly toward the T3b stage (75.5%). It is evident that the effect of CRT is more pronounced in moving cases into the T3b category, although the statistical significance was borderline (P = 0.060). There was a noticeable downstaging after treatment when considering the pathological analyses for both groups. This is particularly significant in the CRT group, which was 67.5% at the T3 stage after treatment. In this study, sampling reduced the impact of MRI T-staging errors in evaluations, thus offering more assurance that instances initiated as T3 and any observed pT-downstaging in histopathological examinations were authentic.

Many studies have shown that after neoadjuvant therapy, the count of lymph nodes typically reduces, and the short-axis diameter either diminishes or vanishes entirely; this shows nodal downstaging [24]. Most of our study patients in the CT group (67.5%) before treatment were staged at N2. Post-treatment MRI showed that the N2 percentage decreased to 35%, whereas N0 increased to 37.5%, signifying effective nodal clearance or downstaging. Pathological staging was consistent, with a majority (52.5%) being staged N0, reinforcing the efficacy of CT in nodal downstaging or clearance. The pre-treatment CRT group had a large portion (63%) of lymph nodes staged at N2. Post-treatment MRI, the N2 group significantly reduced to 6.1% and N0 rose to 38.8%. Again, the post-treatment pathological findings supported this trend, with 67.3% staged as N0. For both treatment modalities, significant nodal downstaging was evident (P = 0.001). Borgheresi et al. [11] concluded that MRI is the primary imaging technique suggested for lymph node assessment in rectal cancer. However, radiologists must understand that MRI's specificity in identifying lymph node metastases is limited, even when integrating size with additional parameters such as DWI/ADC.

While both treatment modalities demonstrated efficacy in tumor and nodal downstaging, FOLFOX CT seemed to have a slightly more diversified post-treatment tumor stage distribution than CRT. Additionally, both treatment modalities showed significant nodal downstaging, with CRT having a slight edge in moving more patients to N0 staging, as observed in both MRI and pathological findings.

The mean ADC increased after treatment in both groups, indicating a possible reduction in tumor cellular density. The ROC curve for rectal cancer detection with ADC showed good sensitivity and specificity (81.8% and 71.4%, respectively), suggesting that ADC may be a reliable metric for rectal cancer characterization. Similarly, the mean ADC values for lymph nodes increased after treatment in both groups. The ROC curve for lymph node detection showed a slightly lower AUC than that for tumor detection, but still had reasonable sensitivity and specificity (78% and 57%, respectively). Recent research suggests a stronger statistical correlation and consensus in the DWI stage compared with the MRI stage when diagnosing rectal carcinoma. This is consistent with the assertion by Schurink et al. [22] that the qualitative and quantitative insights provided by DWI have superior diagnostic value, making it an important complementary tool for the detection of rectal cancer.

The presence of nodal metastases is a critical indicator of both the likelihood of local recurrence and survival without the disease. Therefore, it is important to accurately determine whether neoadjuvant chemotherapy and radiation are required prior to surgery. Some previous studies, such as Li et al. [25], found that the ADC difference value was an optimal parameter for identifying lymph node metastases, lymphovascular invasion, and histology type. In contrast, in our study, there was no significant correlation between the minimum, average, or maximum ADC values of the tumor and the ADC values of lymph nodes. This may imply the inherent heterogeneity between primary tumors and lymphatic metastases or that the mechanisms affecting tumor and nodal responses might differ.

In this study, we attempted to understand the correlation between the ADC values of the tumor, especially the minimum, average, and maximum, and the ADC values of the lymph nodes. Our results showed that there was no significant correlation between these values (P > 0.376). This lack of correlation highlights the possible independence of these two measures in this particular context. Interestingly, the pre-treatment ADC average of what was considered a "healthy" inguinal lymph node/IN was significantly higher in the CT group than in the N ADC group, a difference that was statistically significant (P = 0.001). However, this disparity seemed to diminish after the treatment, reaching a point where no significant difference was observed (P = 0.313). Conversely, for the CRT group, the average ADC of the "healthy" inguinal lymph node/IN remained consistently higher both before and after treatment (P = 0.001), suggesting a more stable or inherent characteristic in this cohort. Such discrepancies between the CT and CRT groups warrant further investigation to understand the underlying dynamics and their potential implications for patient management and predicting treatment outcomes.

However, from another perspective, in the CT group, pre-treatment "healthy" inguinal lymph node ADC values were significantly higher than those with nodal involvement (P = 0.001), although this difference diminished post-treatment. In the CRT group, healthy inguinal lymph nodes maintained significantly higher ADC values both pre- and post-treatment, suggesting a persistent distinction between healthy and involved nodes despite treatment. One of the latest represented studies by Osman et al. concluded that MRI functional imaging, using ADC values can be used to differentiate between metastatic and non-metastatic lymph nodes in rectal adenocarcinoma with a diagnostic accuracy of 86.52% [26].

## Conclusions

Both FOLFOX CT and CRT have demonstrated significant efficacy in the downstaging of rectal tumors and lymph nodes. The lack of correlation between tumor and nodal ADC values suggests that individualized assessments may be essential. The difference in ADC values of "healthy" inguinal lymph nodes in the CRT group may help differentiate involved from non-involved nodes. As much research has investigated the potential of ADC to differentiate between metastatic and non-metastatic nodes in primary rectal cancer, DWIs have recently become an important imaging modality for detecting nodal metastases.
